# Screening for anxiety disorders in patients with coronary artery disease

**DOI:** 10.1186/1477-7525-11-37

**Published:** 2013-03-11

**Authors:** Adomas Bunevicius, Margarita Staniute, Julija Brozaitiene, Victor JM Pop, Julius Neverauskas, Robertas Bunevicius

**Affiliations:** 1 Behavioral Medicine Institute, Lithuanian University of Health Sciences, Palanga, Lithuania; 2 Department of Neurology, University of North Carolina at Chapel Hill, Chapel Hill, NC, USA; 3 CoRPS—Center of Research on Psychology in Somatic diseases, Department of Medical and Clinical Psychology, Tilburg University, Tilburg, The Netherlands

**Keywords:** Coronary artery disease, Anxiety, Screening, Sensitivity, Specificity

## Abstract

**Background:**

Anxiety disorders are prevalent and associated with poor prognosis in patients with coronary artery disease (CAD). However, studies examining screening of anxiety disorders in CAD patients are lacking. In the present study we evaluated the prevalence of anxiety disorders in patients with CAD and diagnostic utility of self-rating scales for screening of anxiety disorders.

**Methods:**

Five-hundred and twenty-three CAD patients not receiving psychotropic treatments at initiation of rehabilitation program completed self-rating scales (Hospital Anxiety and Depression Scale or HADS; Spielberger State-Anxiety Inventory or SSAI; and Spielberger Trait-Anxiety Inventory or STAI) and were interviewed for generalized anxiety disorder (GAD), social phobia, panic disorder and agoraphobia (Mini-International Neuropsychiatric Interview or MINI).

**Results:**

Thirty-eight (7%) patients were diagnosed with anxiety disorder(s), including GAD (5%), social phobia (2%), agoraphobia (1%) and panic disorder (1%). Areas under the ROC curve of the HADS Anxiety subscale (HADS-A), STAI and SSAI for screening of any anxiety disorder were .81, .80 and .72, respectively. Optimal cut-off values for screening of any anxiety disorders were ≥8 for the HADS-A (sensitivity = 82%; specificity = 76%; and positive predictive value (PPV) = 21%); ≥45 for the STAI (sensitivity = 89%; specificity = 56%; and PPV = 14%); and ≥40 for the SSAI (sensitivity = 84%; specificity = 55%; PPV = 13%). In a subgroup of patients (n = 340) scoring below the optimal major depressive disorder screening cut-off value of HADS-Depression subscale (score <5), the HADS-A, STAI and SSAI had moderate-high sensitivity (range from 69% to 89%) and low PPVs (≤22%) for GAD and any anxiety disorders.

**Conclusions:**

Anxiety disorders are prevalent in CAD patients but can be reliably identified using self-rating scales. Anxiety self-rating scales had comparable sensitivities but the HADS-A had greater specificity and PPV when compared to the STAI and SSAI for screening of anxiety disorders. However, false positive rates were high, suggesting that patients with positive screening results should undergo psychiatric interview prior to initiating treatment for anxiety disorders and that routine use of anxiety self-rating scales for screening purposes can increase healthcare costs. Anxiety screening has incremental value to depression screening for identifying anxiety disorders.

## Introduction

Anxiety disorders affect up to 20% of patients across different stages of coronary artery disease (CAD) [1-4]. Generalized anxiety disorder (GAD) is the most prevalent anxiety disorder with point prevalence rates ranging from 5% [1,2] to 12% [3-5]. Other anxiety disorders are less common in CAD patients [2-4]. Although, anxiety, when compared to depression, has received significantly less attention in CAD patients, but emerging data suggest that anxiety disorders are associated with increased risk for all-cause mortality and major adverse cardiac events independently from disease severity, depression and adverse health behaviors [1,2,5]. Anxiety symptoms also predict poor clinical [6,7] and patient–centered outcomes [8,9].

The American Heart Association has recently recommended routine screening for depression in CAD patients [10]. Screening for anxiety disorders was not included in these recommendations despite the fact that anxiety disorders are often under-recognized and untreated in cardiac patients [11-13]. For example, a study in 74 patients admitted for acute myocardial infarction (MI) within 72 hours after symptom onset found that healthcare providers failed to identify 69% of patients with elevated symptoms of anxiety and 50% of patients with anxiety disorders [11]. Another study in 158 stable heart failure patients found that 58% and 60% of patients with positive screening results for depression and/or anxiety disorder(s) on a telephone interview had a documented diagnosis of depression and/or anxiety and received mental health treatment, respectively [12]. On the other hand, up to 92% of patients with a documented diagnosis of depression or anxiety received mental health treatment, suggesting that identification of psychiatric disorders in CAD patients improves mental health treatment availability that could potentially improve prognosis and quality of life of CAD patients [12]. Poor identification of anxiety disorders can be attributed to the fact that healthcare providers are more concerned with management of physical symptoms and rarely used standardized screening scales [13]. Furthermore, data regarding psychometric properties of anxiety self-rating scales remain limited in CAD patients [13].

We and others have previously shown that Depression subscale of the Hospital Anxiety and Depression Scale (HADS-D) [14] and Beck Depression Inventory-II [15] had acceptable psychometric properties for screening of depressive disorders and for evaluation of depressive symptom severity across different stages of CAD [16-19]. Recently, Frasure-Smith and Lesperance demonstrated that Anxiety subscale of the HADS (HADS-A) had acceptable sensitivity and specificity for screening of generalized anxiety disorder (GAD) in stable CAD patients two months after acute coronary syndromes [2]. The Spielberger State-Trait Anxiety Inventory was designed to evaluate anxiety as personality trait (Spielberger Trait Anxiety Inventory or STAI) and as emotional state (Spielberger State Anxiety Inventory or SSAI) [20]. In CAD patients, the STAI and SSAI are widely used for evaluation of anxiety symptom severity but screening properties of these instruments remain to be investigated [21,22].

The aims of this study were to establish the prevalence of anxiety disorders in stable CAD patients undergoing rehabilitation, and to evaluate the internal consistency and psychometric properties of the HADS-A, STAI and SSAI for screening of anxiety disorders.

## Methods

### Patients and procedure

In the period from October 2007 until December 2011 consecutive patients attending a cardiac rehabilitation program at the Behavioral Medicine Institute of the Lithuanian University of Health Sciences in Palanga, Lithuania were considered for this study. In Lithuania, patients are referred for cardiac rehabilitation within one week after discharge from cardiology inpatient unit following treatment for acute coronary syndromes. Hence, patients were included in this study approximately two weeks after experiencing an episode of acute coronary syndrome. Patients were not invited to the study if they were older than 80 years of age, had severe medical co-morbidities, unstable cardiovascular status, communication problems or did not speak Lithuanian fluently. A total of 648 patients met the study criteria and agreed to participate in the study. However, for the purpose of the present report and in accordance with recent recommendation for diagnostic accuracy studies of depression scales, 125 (19%) patients receiving treatment with psychotropic medication were not included in the analyses [23]. Thus, our final study sample consisted of 523 patients. Data on personality related differences and on evaluation of depression in this cohort of patients were previously published elsewhere [17,24-28].

Within three days of admission all patients were evaluated by study cardiologists for demographic characteristics, New York Heart Association (NYHA) functional class [29], hypertension, angina pectoris [30], obesity, previous interventional treatments for CAD (percutaneous coronary intervention (PCI) or coronary artery bypass graft surgery), current use of cardiac and psychotropic medication, and histories of MI and diabetes mellitus. Hypertension was defined as systolic blood pressure ≥ 140 mmHg and/or diastolic blood pressure ≥ 90 mmHg. Patients with body mass index ≥ 30 kg/m^2^ were considered obese. During the same visit patients were evaluated for anxiety symptoms and depression using the HADS [14], STAI, SSAI [20] and BDI-II [15]. Scales were checked for missing items and patients were asked to complete the missing items. On the next day patients were interviewed for current psychiatric disorders according to the Diagnostic and Statistical Manual of Mental Disorders, 4th edition, Text revision (DSM-IV-TR) criteria using the Mini International Neuropsychiatric Interview (MINI) [31-33]. The interviewer was blind to clinical characteristics, psychiatric histories, psychotropic treatments and scores on anxiety and depression self-rating scales. The interviewer was trained to administer the MINI by study psychiatrist over two 120-minutes training sessions. Two unclear diagnostic cases were discussed with the training psychiatrist and included the differentiation of GAD and major depressive episode (MDE) from adjustment disorder due to acute cardiac event.

The study and its consent procedures were approved by the Ethics Committee for Biomedical Research at the Lithuanian University of Health Sciences. A written informed consent was obtained from each study patient.

### Instruments

Diagnosis of anxiety disorder according to the MINI was considered the gold standard [33]. Patients were interviewed using all modules of the MINI, except for antisocial personality disorder. For purposes of this study, we used modules of the MINI pertaining current GAD, current social phobia, current panic disorder, current agoraphobia and current MDE. Patients were not interviewed for histories of anxiety disorders. Each module of the MINI consists of a screening question that is followed by diagnostic questions. Administration of the MINI takes from 2 to 20 minutes, depending on presence and complexity of psychiatric disorder(s). As per DSM-IV-TR criteria and MINI instructions, patients with current MDE were not evaluated for GAD if symptoms of anxiety were limited exclusively to the depressive episode. It is well documented that non-English translations of the MINI, including Japanese [34], French [35] and Portuguese [36], have acceptable validity against other structured psychiatric interviews, including the Structured Diagnostic Interview for DSM (SCID) [37]. Lithuanian version of the MINI was established by using double-blind translation method and was approved by the authors who developed the original version of the MINI [32]. The MINI is widely used in commercial clinical trials and for research purposes in Lithuania [17,38,39].

The HADS is comprised of two 7-item subscales of depression (HADS-D) and anxiety (HADS-A) designed to measure respective symptoms during the past week. Each HADS item is rated from 0 to 3, with total score ranging from 0 to 21 and with higher score indicating more severe anxiety symptoms. It was originally proposed that scores from 8 to 10 suggest, and scores ≥ 11 indicate probable anxiety and depressive disorder [14]. The HADS-A evaluates symptoms of GAD and panic disorder [14]. The HADS is well-validated in Lithuania for screening of anxiety disorders and MDE in primary care patients [38]. We have recently shown that the HADS-D had adequate psychometric properties at a cut-off value of ≥5 for screening of MDE in CAD patients [17]. Acceptable internal consistency (Cronbach’s coefficient alpha = .83) and high sensitivity (91%) of the HADS-A at a cut-off score of ≥8 for GAD were recently reported in post-MI patients [2].

The Spielberger State-Trait Anxiety Inventory [20] is comprised of two self-rating questionnaires: the SSAI and STAI. Each questionnaire contains 20 statements that are rated on 4-point Likert type scale. The SSAI is a measure of how the patient is feeling at the particular moment (state), whereas the STAI is a measure of the patient’s general level of anxiety (trait). SSAI and STAI items are divided into two groups: 10 items and 13 items, respectively, are formed to record the presence of anxiety symptoms or traits and remaining items are designed to record the absence of anxiety symptoms or traits and are reverse scored [20]. Higher scores on both scales indicate more anxiety symptoms. Scores on the STAI and SSAI ≥ 30 suggest moderate anxiety and scores ≥ 45 suggest severe anxiety [20]. Lithuanian translations of the SSAI and STAI are used for research purposes for at least two decades in Lithuania [40].

The BDI-II is a 21-item self-rating scale that evaluates for depressive symptom severity during the previous 2 weeks [15]. We have recently demonstrated the BDI-II cut-off score of ≥14 had optimal psychometric properties for screening of MDE in CAD patients undergoing rehabilitation [17].

### Statistical analyses

First, we examined psychometric properties of the HADS-A, SSAI and STAI for screening of GAD only and for any anxiety disorders. Patients were assigned to any anxiety disorders group if they were positive for current DSM-IV-TR diagnoses of GAD, social phobia, panic disorder and/or agoraphobia on the MINI. We performed receiver operating characteristics (ROC) curve analyses and calculated the areas under the ROC curve (AUCs). Cut-off values with optimal balance between sensitivity and specificity were determined by visually assessing the ROC curves. For each optimal cut-off value we computed sensitivity (true-positive rate), specificity (true-negative rate), positive predictive value (PPV; proportion of patients with positive test results who were correctly diagnosed), negative predictive value (NPV; proportion of patients with negative test results who were correctly diagnosed) and accuracy. We also calculated 95% Confidence Intervals (CI) for each value of sensitivity, specificity, PPV and NPV.

Next, we investigated how much screening for anxiety disorders added incrementally to depression screening. For these analyses, we excluded patients who screened positive for MDE using previously reported optimal cut-off values of the HADS-D (≥5) and BDI-II (≥14) [17]. Subsequently, in the latter subgroups of patients, we investigated sensitivities, specificities, PPVs and NPVs of the HADS-A, STAI and SSAI at optimal-cut off values for screening of GAD and any anxiety disorders.

Data were analyzed using the PASW for Windows (IBM Corporation, Chicago, Illinois). Data are expressed as mean ± standard deviation and as median (interquartile range; IQR) for quantitative variables, and as number (percent) for qualitative variables.

## Results

### Baseline characteristics

Demographic, clinical and psychiatric characteristics of 523 study patients are presented in Table 1. Mean age of study patients was 57.5 ± 9.2 years. The majority of patients were men (78%), were NYHA functional class II (76%), had hypertension (80%), angina pectoris (59%), history of acute MI (69%) and previous PCI (78%). Nine percent of patients had diabetes and 47% were obese.

**Table 1:** Demographic, clinical and psychiatric characteristics of 523 patients with coronary artery disease

**Demographic and clinical characteristics**	
Age, years, mean ± SD, median, IQR	57.5 ± 9.2; 57; 13
Gender, n (%)	
Men	407 (78)
Women	116 (22)
NYHA class, n (%)	
I	46 (9)
II	397 (76)
III	80 (15)
Hypertension, n (%)	419 (80)
Angina pectoris, n (%)	306 (59)
History of myocardial infarction, n (%)	363 (69)
Previous treatments, n (%)	
Percutaneous coronary intervention	409 (78)
Coronary artery by-pass graft surgery	13 (3)
Diabetes mellitus, n (%)	45 (9)
Body mass index ≥30 kg/m^2^, n (%)	247 (47)
**Psychiatric characteristics**	
Current MINI anxiety diagnoses, n (%):	
Generalized anxiety disorder	26 (5)
Agoraphobia	7 (1)
Panic disorder	4 (1)
Social phobia	8 (2)
Any anxiety disorder	38 (7)
Any anxiety disorder plus MDE	10/38 (26)
HADS-A score, mean ± SD, median, IQR	5.7 ± 3.6; 5; 5
STAI score, mean ± SD, median, IQR	44.1 ± 10.1; 44; 14
SSAI score, mean ± SD, median, IQR	39.0 ± 10.4; 39; 13
HADS-D score ≥5	183 (35)
BDI-II score ≥14	153 (29)

*HADS-A* - Anxiety subscale of the Hospital Anxiety and Depression Scale; *HADS-D* - Depression subscale of the Hospital Anxiety and Depression Scale; *BDI-II* – Beck Depression Inventory-II; *MDE* – major depressive episode; *IQR*- interquartile range; *NYHA* - New York Heart Association; *SSAI* - Spielberger State Anxiety Inventory; *STAI* - Spielberger State Anxiety Inventory.

Thirty-eight (7%) patients were diagnosed with any anxiety disorder. The most prevalent anxiety disorder was GAD (5%), followed by social phobia (2%), agoraphobia (1%) and panic disorder (1%). Thirty five percent and 29% of patients were screened positive for MDE according to the HADS-D (score ≥5) and BDI-II (score ≥14), respectively.

### Screening for anxiety disorders

AUCs for the HADS-A, STAI and SSAI for screening of GAD were at levels of .85, .82 and .74, respectively (Table 2). Detailed psychometric properties of the HADS-A, STAI and SSAI at optimal cut-off values for screening of GAD are presented in Table 2. Specifically, optimal cut-off values for screening of GAD were ≥8 for the HADS-A (sensitivity = 92%, specificity = 75% and PPV = 16%), ≥45 for the STAI (sensitivity = 92%, specificity = 55% and PPV = 10%) and ≥40 for the SSAI (sensitivity = 89%, specificity = 54% and PPV = 9%).

**Table 2:** Psychometric properties at optimal cut-off values of self-rating anxiety scales for screening of generalized anxiety disorder in coronary artery disease patients (n = 523)

**Cut-off scores**	**N (%)**	**Sensitivity,% (95% CI)**	**Specificity,% (95% CI)**	**PPV,% (95% CI)**	**NPV,% (95% CI)**	**Accuracy,%**	**AUC (95% CI)**
Hospital anxiety and depression scale – anxiety subscale							
≥ 7	203 (39)	92 (73–99)	64 (60–68)	12 (8–17)	99 (98–100)	65	.85 (.76-.93)
≥ **8**	148 (28)	92 (73–99)	75 (71–79)	16 (11–23)	99 (98–100)	76	
≥ 9	100 (19)	69 (48–85)	84 (80–87)	18 (11–27)	98 (96–99)	83	
Spielberger trait anxiety inventory							
≥ 44	262 (50)	92 (73–99)	52 (48–57)	9 (6–13)	99 (97–100)	54	.82 (.75-.89)
≥ **45**	246 (47)	92 (73–99)	55 (51–60)	10 (6–14)	99 (97–100)	57	
≥ 46	229 (44)	85 (64–95)	58 (54–63)	10 (6–14)	99 (96–100)	60	
Spielberger state anxiety inventory							
≥ 39	267 (51)	89 (69–97)	51 (46–55)	9 (6–13)	99 (96–100)	53	.74 (.66-.82)
≥ **40**	251 (48)	89 (69–97)	54 (50–59)	9 (6–14)	99 (97–100)	56	
≥ 41	226 (43)	81 (60–93)	59 (54–63)	9 (6–14)	98 (96–99)	60	

Optimal cut-off value in **bold.**

*AUC* - area under the receiver operating curve; *CI* – confidence interval; *NPV*- negative predictive value; *PPV* - positive predictive value.

With regards to screening of any anxiety disorders, the AUCs for the HADS-A, STAI and SSAI were at levels of .81, .80 and .72, respectively (Table 3). Table 3 demonstrates that optimal cut-off values for screening of any anxiety disorder were the same as for screening of GAD only. Specifically, optimal cut-off values were ≥8 for the HADS-A (sensitivity = 82%, specificity = 76% and PPV = 21%), ≥45 for the STAI (sensitivity = 89%, specificity = 56% and PPV = 14%) and ≥40 for the SSAI (sensitivity = 84%, specificity = 55% and PPV = 13%). Sensitivities and specificities of anxiety self-rating scales across different cut-off values for screening of GAD and any anxiety disorder are presented in Additional file 1: Table S1 and S2.

**Table 3:** Psychometric properties at optimal cut-off values of self-rating anxiety scales for screening of any anxiety disorders in coronary artery disease patients (n = 523)

**Cut-off score**	**N (%)**	**Sensitivity,% (95% CI)**	**Specificity,% (95% CI)**	**PPV,% (95% CI)**	**NPV,% (95% CI)**	**Accuracy,%**	**AUC (95% CI)**
Hospital anxiety and depression scale – anxiety subscale							
≥ 7	203 (39)	87 (71–95)	65 (60–69)	16 (12–22)	98 (96–99)	67	.81 (.74-.89)
≥ **8**	148 (28)	82 (65–92)	76 (72–80)	21 (15–29)	98 (96–99)	76	
≥ 9	100 (19)	66 (49–80)	85 (81–88)	25 (17–35)	97 (95–98)	83	
Spielberger trait anxiety inventory							
≥ 44	262 (50)	89 (74–97)	53 (48–57)	13 (9–18)	98 (96–100)	53	.80 (.73-.87)
≥ **45**	246 (47)	89 (74–97)	56 (52–61)	14 (10–19)	99 (96–100)	56	
≥ 46	229 (44)	84 (68–93)	59 (55–64)	14 (10–19)	98 (95–99)	59	
Spielberger state anxiety inventory							
≥ 39	267 (51)	84 (68–93)	52 (47–56)	12 (8–17)	98 (95–99)	54	.72 (.64-.79)
≥ **40**	251 (48)	84 (68–93)	55 (50–59)	13 (9–18)	98 (95–99)	57	
≥ 41	226 (43)	76 (59–88)	59 (55–64)	13 (9–18)	97 (94–99)	61	

Optimal cut-off value in **bold.**

*AUC* - area under the receiver operating curve; *CI* – confidence interval; *NPV*- negative predictive value; *PPV* - positive predictive value.

### Incremental value of anxiety screening

In a subgroup of patients who scored <5 on the HADS-D (i.e., screened negative for MDE; n = 340), the HADS-A, STAI and SSAI at optimal cut-off values had moderate to high sensitivity (range from 69% to 89%) and specificity (range from 67% to 88%) but low PPVs (range from 7% to 22%) for GAD and any anxiety disorders (Table 4). Similarly, in a subgroup of patients screened negative for MDE according to the BDI-II (score <14; n = 370), the HADS-A, STAI and SSAI had moderate to high sensitivity (range from 50% to 80%) and specificity (range from 66% to 89%), and low PPVs (range from 2% to 11%) for GAD and any anxiety disorders.

**Table 4:** Psychometric properties at optimal cut-off values for generalized anxiety disorder and any anxiety disorder screening in patients with negative depression screening results

**Cut-off score**	**N (%)**	**Sensitivity,% (95% CI)**	**Specificity,% (95% CI)**	**PPV,% (95% CI)**	**NPV,% (95% CI)**
**HADS-D score <5, n = 340**					
**Generalized anxiety disorder**					
HADS-A ≥ 8	49 (14)	89 (51–99)	88 (83–91)	16 (8–10)	100 (98–100)
STAI ≥ 45	104 (31)	89 (51–99)	71 (66–76)	8 (4–15)	100 (97–100)
SSAI ≥ 40	118 (35)	89 (51–99)	67 (61–72)	7 (3–13)	100 (97–100)
**Any anxiety disorder**					
HADS-A ≥ 8	49 (14)	69 (41–88)	88 (84–91)	22 (12–37)	98 (96–99)
STAI ≥ 45	104 (31)	87 (58–98)	72 (67–77)	13 (71–21)	99 (97–100)
SSAI ≥ 40	118 (35)	81 (54–95)	68 (62–73)	11 (6–18)	99 (96–100)
**BDI-II score <14, n = 370**					
**Generalized anxiety disorder**					
HADS-A ≥ 8	45 (12)	80 (30–99)	89 (85–92)	9 (3–22)	100 (98–100)
STAI ≥ 45	110 (30)	60 (17–93)	71 (66–75)	3 (1–8)	99 (97–100)
SSAI ≥ 40	126 (34)	60 (17–93)	66 (61–71)	2 (1–7)	99 (97–100)
**Any anxiety disorder**					
HADS-A ≥ 8	45 (12)	50 (20–80)	89 (85–92)	11 (4–25)	98 (96–99)
STAI ≥ 45	110 (30)	70 (35–92)	71 (66–76)	6 (3–13)	99 (96–100)
SSAI ≥ 40	126 (34)	50 (20–80)	66 (61–71)	4 (1–9)	98 (95–99)

*BDI-II* – Beck Depression Inventory-II; *CI* – confidence interval; *HADS -A* – Hospital Anxiety and Depression scale – Anxiety subscale; *HADS -D* – Hospital Anxiety and Depression scale – Depression subscale *NPV*- negative predictive value; *PPV* - positive predictive value.

## Discussion

In patients with CAD undergoing cardiac rehabilitation, the prevalence of anxiety disorders, especially GAD, was high. Anxiety self-rating scales had comparable sensitivities, but the HADS-A had better specificities for screening of GAD and any anxiety disorders when compared with the STAI and SSAI. However, positive predictive values were low of all anxiety self-rating scales analyzed in the present study. Addition of anxiety screening to depression screening had incremental value for identification of anxiety disorders.

Prevalence of anxiety disorders in our cohort corresponds to previous studies. For example, similar prevalence rate of anxiety disorders was reported by Frasure-Smith and Lesperance in stable post-MI patients [2]. Specifically, they reported that 5% of their patients had current GAD; 2% had panic disorder and <1% had social phobia according to the Structured Clinical Interview for the DSM [2,41]. Another recent study found that 10% of stable CAD patients met GAD criteria in the past year according to the Diagnostic Interview Schedule for the DSM-IV [5]. Parker with colleagues, in patients with acute coronary syndromes, found higher prevalence rates of GAD (12%) and social phobia (9%) and similar prevalence rates of agoraphobia (2%) and panic disorder (2%) according to the Composite International Diagnostic Interview [3,42]. In patients awaiting coronary revascularization procedure, Tully and Penninx also reported greater prevalence rates of MINI diagnoses of GAD (10%), agoraphobia (4%) and social phobia (3%) [4]. These data suggest that the prevalence of anxiety disorders can be different across different populations of CAD patients. Hence, large epidemiological studies evaluating the prevalence of anxiety disorders in CAD patients diagnosed using well-validated and reliable instruments are needed.

The HADS-A had superior psychometric properties for screening of GAD and any anxiety disorders when compared to the STAI and SSAI. Specifically, although at optimal cut-off values all three anxiety scales had similar sensitivities for screening of GAD and any anxiety disorders, but the HADS-A had greater specificities and PPVs when compared with the STAI and SSAI. However, confidence intervals for sensitivities were wide and can be explained by small numbers of cases. Hence, further studies in larger samples of CAD patients and meta-analysis of currently published anxiety screening studies in CAD patients should be undertaken in order to provide with more reliable estimates of optimal cut-off values of anxiety screening scales.

At cut-off value of ≥ 8 the HADS-A yielded optimal psychometric properties for screening of GAD and any anxiety disorders. Similar sensitivity (91%) and lower specificity (61%) of the HADS-A at cut-off value of ≥8 for GAD was previously reported in stable CAD patients [2]. Marginal differences in psychometric properties of the HADS-A in the latter study when compared to our results can be partially explained by different gold standards used and by different time-frame of psychiatric evaluation with respect to acute coronary syndromes. Furthermore, in our study, at cut-off value of ≥8 the HADS-A had acceptable psychometric properties for screening of any anxiety disorder and GAD only, suggesting that CAD patients scoring ≥8 on the HADS-A should undergo psychiatric evaluation for all anxiety disorders, rather than for GAD alone.

Optimal cut-off values of the STAI and SSAI were ≥45 and ≥40, respectively, for GAD and for any anxiety disorder. A recent study in 100 pregnant women reported optimal cut-off value of the STAI and SSAI of ≥ 40 with sensitivities and specificities of about 80% for both scales for screening of current MINI diagnoses of panic disorder, agoraphobia, social phobia, GAD, and posttraumatic stress disorder [43]. Lower sensitivities and greater specificities of the STAI and SSAI in pregnant women can be attributed to different patients’ populations and to different anxiety disorders evaluated across studies. Nonetheless, to the best of our knowledge, ours was the first study evaluating psychometric properties of the SSAI and STAI for screening for current anxiety disorders in CAD patients and our results remain to be replicated in independent samples of CAD patients.

It should be noted that the HADS-A, STAI and SSAI had low PPVs, suggesting that these scales are overly inclusive and the majority of CAD patients with positive screening results will not meet the DSM-IV-TR criteria for anxiety disorders. A study by Frasure-Smith and Lesperance in post-MI patients, found similar PPV (12%) of the HADS-A for screening of GAD [2]. Also, low PPVs of the HADS-D for screening of major depressive disorder were previously reported in CAD patients [17,18]. These findings suggest that somatic symptoms can interfere with subjective evaluation of mental distress using self-rating scales and that significant proportion of CAD patients suffer from mental distress that does not reach the DSM-IV-TR severity threshold criteria [44,45]. Low PPVs of anxiety self-rating-scales can be partially explained by low prevalence rate of anxiety disorders and by possible residual and/or subclinical symptoms of mood or anxiety disorders in the subgroup of patients without current anxiety disorders. However, anxiety prevalence rates in our cohort correspond to the existing literature and subclinical symptoms of psychological distress are highly prevalent in CAD patients. The major goal of anxiety screening is differentiation of patients with anxiety disorder(s) from those with subclinical symptoms of psychological distress.

High false-positive rates suggest that mental health treatment should not be initiated based solely on screening results and patients with positive screening results should be always referred for detailed psychiatric evaluation by mental health specialists. High false positive rates also indicate that routine use of the HADS-A, STAI and SSAI for anxiety disorder screening purposes can overstretch healthcare resources. On the other hand, high specificities and high NPVs of the HADS-A, STAI and SSAI suggest that anxiety disorders are highly unlikely among CAD patients scoring below the recommended threshold values.

Our results also provided with evidence that anxiety screening can have incremental value to depression screening. Specifically, we found that depression screening can miss a substantial proportion of patients suffering from anxiety disorder, but these patients can be identified with anxiety screening. A two-step diagnostic algorithm can be particularly useful in the light of high false positive rates, is currently recommended for depression screening [10] and should be investigated for anxiety screening purposes in CAD patients.

There remains a debate in the literature whether there is enough evidence to justify systematic screening for mental disorders in CAD patients. Specifically, although systematic screening for depression has been recommended in CAD patients [10], but these guidelines were challenged mainly because there is not a single randomized controlled trial demonstrating benefits of such screening for clinical and mental health outcomes [23,46-49]. In addition, costs and safety of depression screening warrants additional research [23,47]. Similar limitations apply to anxiety disorder screening, since there are no studies demonstrating clinical benefits, safety and cost-effectiveness of such practice. Thus, prior to recommending routine screening of anxiety disorders in CAD patients there is a need for well conducted randomized trials clearly demonstrating that systematic anxiety screening is safe, cost-effective and carries clear clinical benefits, such as reduction of anxiety symptoms and improved clinical outcomes.

Exclusion of patients with advanced age, severe co-morbidities or unstable cardiovascular status can limit generalizability of our results. Also, our results cannot be applied to patients with acute coronary syndromes because we studied stable CAD patients admitted for cardiac rehabilitation. In addition, future studies should consider detailed evaluation for psychiatric histories since residual symptoms of mood and anxiety disorders in patients without current anxiety disorder can potentially impact the screening results. Finally, test-retest reliability of anxiety self-rating scales was not investigated. However, rehabilitation offers excellent opportunity for identification of mental distress when there are no life-threats that could potentially intervene with psychological assessments. The major strengths of our study include large sample size and the use of structured clinical psychiatric interview.

## Conclusions

In sum, anxiety disorders are prevalent in CAD patients and can be reliably identified using self-rating scales. All anxiety self-rating scales analyzed in the present report have similar sensitivities, but the HADS-A has superior specificities and PPVs when compared to the STAI and SSAI for screening of anxiety disorder in CAD patients undergoing rehabilitation. However, patients with positive screening results should undergo comprehensive psychiatric evaluation in order to establish diagnosis and initiate optimal treatment strategy. Anxiety screening has incremental value to depression screening for identifying anxiety disorders. Finally, studies demonstrating safety, cost-effectiveness and clinical benefits of anxiety screening are warranted prior to recommending routine screening of anxiety disorders outside of research protocols.

## Competing interests

The authors declare that they have no competing interests.

## Authors’ contributions

AB undertook statistical analyses, interpretation of findings and wrote the first draft of the manuscript. MS, JB and JN contributed to preparation of study protocol and were involved in the management of study protocol, data collection and management of the database. VJMP reviewed and commented on drafts, provided advice regarding interpretation of the data. RB designed the study, wrote the protocol, contributed to interpretation of findings and reviewed all drafts. All authors read and approved the final manuscript.

## Supplementary Material

Additional file 1: Table S1 “Psychometric properties of the HADS across 18 cut-off values”; **Table S2.** “Psychometric properties of the STAI and SSAI across 30 cut-off values”. Click here for file
