# Supplementary material for: Screening for anxiety disorders in patients with coronary artery disease
Source: Health Qual Life Outcomes. 2013 Mar 11;11:37. doi: 10.1186/1477-7525-11-37 (PMC3601013; doi:10.1186/1477-7525-11-37)
Supplement: Additional file 1: Table S1 — “Psychometric properties of the HADS across 18 cut-off values”; Table S2. “Psychometric properties of the STAI and SSAI across 30 cut-off values”. [file 1477-7525-11-37-S1.doc]

**Supplementary tables**

Table 1. Sensitivity and specificity of the Hospital Anxiety and Depression Scale – Anxiety subscale across 18 cut-off values for screening of generalized anxiety disorder and any anxiety disorders (n=523).

|  | Generalized anxiety disorder | | Any anxiety disorder | |
| --- | --- | --- | --- | --- |
| HADS-A cut-off score | Sensitivity (%) | Specificity (%) | Sensitivity (%) | Specificity (%) |
| ≥1 | 100 | 5 | 97 | 5 |
| ≥2 | 100 | 11 | 97 | 11 |
| ≥3 | 92 | 20 | 92 | 21 |
| ≥4 | 92 | 34 | 92 | 34 |
| ≥5 | 92 | 44 | 89 | 45 |
| ≥6 | 92 | 54 | 89 | 55 |
| ≥7 | 92 | 64 | 87 | 65 |
| ≥8 | 92 | 75 | 82 | 76 |
| ≥9 | 69 | 84 | 66 | 85 |
| ≥10 | 54 | 90 | 47 | 90 |
| ≥11 | 50 | 92 | 39 | 92 |
| ≥12 | 38 | 95 | 32 | 95 |
| ≥13 | 27 | 96 | 21 | 96 |
| ≥14 | 23 | 98 | 18 | 98 |
| ≥15 | 12 | 98 | 11 | 99 |
| ≥16 | 8 | 99 | 8 | 99 |
| ≥17 | 4 | 99 | 5 | 100 |
| ≥18 | 0 | 100 | 3 | 100 |

HADS-A, Hospital Anxiety and Depression Scale – Anxiety subscale.

Table 2. Sensitivity and specificity of the Spielberger Trait Anxiety Inventory and Spielberger State Anxiety Inventory across 30 cut-off values for screening of generalized anxiety disorder and any anxiety disorders (n=523).

|  | Spielberger Trait Anxiety Inventory | | | | Spielberger State Anxiety Inventory | | | |
| --- | --- | --- | --- | --- | --- | --- | --- | --- |
|  | Generalized anxiety disorder | | Any anxiety disorder | | Generalized anxiety disorder | | Any anxiety disorder | |
|  | Sens. (%) | Spec. (%) | Sens. (%) | Spec. (%) | Sens. (%) | Spec. (%) | Sens. (%) | Spec. (%) |
| ≥30 | 100 | 8 | 97 | 8 | 100 | 19 | 97 | 20 |
| ≥31 | 100 | 9 | 97 | 9 | 100 | 23 | 97 | 23 |
| ≥32 | 100 | 12 | 97 | 12 | 100 | 26 | 97 | 26 |
| ≥33 | 100 | 14 | 97 | 14 | 96 | 28 | 95 | 28 |
| ≥34 | 100 | 17 | 97 | 17 | 96 | 31 | 95 | 31 |
| ≥35 | 100 | 19 | 97 | 19 | 92 | 34 | 92 | 34 |
| ≥36 | 100 | 22 | 97 | 22 | 92 | 38 | 92 | 38 |
| ≥37 | 100 | 25 | 97 | 25 | 92 | 42 | 92 | 43 |
| ≥38 | 100 | 29 | 97 | 29 | 88 | 47 | 84 | 47 |
| ≥39 | 100 | 33 | 95 | 34 | 89 | 51 | 84 | 52 |
| ≥40 | 100 | 37 | 95 | 38 | 89 | 54 | 84 | 55 |
| ≥41 | 96 | 41 | 92 | 42 | 81 | 59 | 76 | 59 |
| ≥42 | 92 | 44 | 89 | 45 | 73 | 64 | 68 | 65 |
| ≥43 | 92 | 46 | 89 | 47 | 65 | 68 | 63 | 69 |
| ≥44 | 92 | 52 | 89 | 53 | 62 | 71 | 55 | 71 |
| ≥45 | 92 | 55 | 89 | 56 | 54 | 74 | 47 | 74 |
| ≥46 | 85 | 58 | 84 | 59 | 42 | 77 | 39 | 77 |
| ≥47 | 85 | 62 | 84 | 63 | 38 | 80 | 34 | 80 |
| ≥48 | 77 | 67 | 74 | 68 | 35 | 82 | 29 | 82 |
| ≥49 | 73 | 71 | 68 | 72 | 35 | 84 | 29 | 84 |
| ≥50 | 73 | 74 | 68 | 74 | 35 | 86 | 29 | 86 |
| ≥51 | 73 | 76 | 68 | 76 | 35 | 88 | 29 | 88 |
| ≥52 | 69 | 78 | 66 | 79 | 31 | 89 | 26 | 89 |
| ≥53 | 65 | 81 | 63 | 82 | 27 | 91 | 24 | 91 |
| ≥54 | 62 | 85 | 61 | 86 | 27 | 93 | 24 | 93 |
| ≥55 | 58 | 86 | 55 | 87 | 27 | 95 | 24 | 95 |
| ≥56 | 54 | 88 | 50 | 89 | 19 | 95 | 18 | 95 |
| ≥57 | 46 | 90 | 45 | 91 | 19 | 96 | 18 | 96 |
| ≥58 | 42 | 91 | 42 | 91 | 15 | 97 | 16 | 97 |
| ≥59 | 35 | 92 | 37 | 93 | 15 | 97 | 16 | 98 |
| ≥60 | 27 | 93 | 32 | 94 | 12 | 97 | 13 | 98 |

Sens. – sensitivity; Spec. – specificity.
